# The Use of Biological Therapies in the Treatment of Chronic Rhinosinusitis with Nasal Polyps: Current State of Knowledge

**DOI:** 10.3390/jcm15155837

**Published:** 2026-07-26

**Authors:** Joanna Wrona, Zuzanna Krupa, Marta Zawadzka, Julia Rydzek, Adrian Muzyka, Karolina Dorobisz, Katarzyna Pazdro-Zastawny

**Affiliations:** 1 Faculty of Medicine, Wroclaw Medical University, Borowska 213, 50-367 Wroclaw, Poland; joanna.wrona@student.umw.edu.pl (J.W.); zuzanna.krupa@student.umw.edu.pl (Z.K.); julia.rydzek@student.umw.edu.pl (J.R.); adrian.muzyka@student.umw.edu.pl (A.M.); 2 Department of Otolaryngology, Head and Neck Surgery, Wroclaw Medical University, Borowska 213, 50-556 Wroclaw, Poland; karolina.dorobisz@umw.edu.pl (K.D.); katarzyna.pazdro-zastawny@umw.edu.pl (K.P.-Z.)

**Keywords:** chronic rhinosinusitis, nasal polyps, biological therapies, monoclonal antibodies, type 2 inflammation, dupilumab, omalizumab, mepolizumab, benralizumab, eosinophils

## Abstract

Chronic rhinosinusitis with nasal polyps (CRSwNP) is a heterogeneous inflammatory disease with a complex pathogenesis that significantly affects patients’ quality of life. Type 2 inflammation plays a dominant role in its course and is associated with the activation of immune pathways involving interleukins IL-4, IL-5 and IL-13, eosinophils, and immunoglobulin E. Standard treatment methods, including corticosteroids and surgical interventions, despite their proven efficacy, often fail to provide sustained disease control and are associated with a high rate of recurrence. The aim of this review is not only to summarize the available evidence on biological therapies in CRSwNP, but also to critically evaluate their current position within treatment algorithms, with particular emphasis on patient selection, integration with endoscopic sinus surgery, comparison of available biologic mechanisms, and remaining challenges in personalized treatment strategies. The paper discusses available monoclonal antibodies, such as dupilumab, omalizumab, mepolizumab, and benralizumab, which act by selectively inhibiting key mediators of type 2 inflammation. Analysis of clinical trial results indicates that biological therapies lead to a significant reduction in nasal polyp size, improvement in nasal patency, restoration of olfactory function, and enhancement of quality of life as measured by the SNOT-22 scale. Furthermore, they demonstrate a favourable safety profile and may represent an effective therapeutic option for patients with severe, treatment-resistant disease, particularly in cases with coexisting eosinophilic asthma. Biological therapies represent a breakthrough in the treatment of CRSwNP and align with the concept of personalised medicine. Their role in clinical practice continues to expand; however, further research is required to optimise patient selection and assess long-term treatment outcomes.

## 1. Introduction

Chronic rhinosinusitis with nasal polyps (CRSwNP) is a form of chronic inflammation of the nasal and paranasal sinus mucosa characterised by the presence of bilateral, benign mucosal growths within the nasal cavity and sinuses [1,2]. According to current diagnostic criteria, symptoms persist for at least 12 weeks and include nasal obstruction, nasal discharge, facial pressure, and olfactory dysfunction [1,2]. Chronic rhinosinusitis (CRS) is classified into two main phenotypes: CRSwNP and chronic rhinosinusitis without nasal polyps (CRSsNP), which differ in both clinical presentation and underlying pathophysiological mechanisms [2,3]. CRSwNP is most commonly associated with type 2 inflammation, whereas CRSsNP more frequently presents a type 1 or mixed inflammatory profile with neutrophilic involvement [2,3].

From an epidemiological perspective, CRS represents a significant public health concern, affecting approximately 5–12% of the general population. The phenotype with nasal polyps occurs in approximately 1–4% of adults and is more common in men [4,5]. Major risk factors include age, male sex, genetic predisposition, asthma, eosinophilia, and environmental exposures [5]. CRSwNP significantly impairs quality of life, with chronic nasal obstruction, loss of smell, and sleep disturbances leading to reduced daily functioning, decreased work productivity, and impaired mental health. The overall burden of the disease is comparable to other chronic conditions such as asthma and diabetes [6,7].

Type 2 inflammation plays a central role in the pathogenesis of CRSwNP, with overproduction of IL-4, IL-5, and IL-13 by Th2 lymphocytes and type 2 innate lymphoid cells (ILC2), leading to eosinophilia, elevated IgE levels, mucosal remodelling, and polyp formation [6,8]. Eosinophils and mast cells, activated by local IgE and epithelial-derived alarmins such as IL-25, IL-33, and TSLP, release inflammatory mediators and leukotrienes that perpetuate chronic inflammation [8,9].

CRSwNP frequently coexists with asthma, allergic rhinitis, and aspirin-exacerbated respiratory disease/NSAID-Exacerbated Respiratory Disease (AERD/NSAID-ERD), which share a common type 2 inflammatory pathway and are associated with a more severe and recurrent disease course [5,6,7].

Diagnosis of CRSwNP is based on the presence of symptoms lasting at least 12 weeks, supported by endoscopic findings and computed tomography (CT) imaging of the sinuses. Disease severity is commonly assessed using tools such as the Lund–Mackay score, Nasal Polyp Score (NPS), and the Sino-Nasal Outcome Test (SNOT-22) [1,10].

Although several randomized controlled trials, systematic reviews, and network meta-analyses have demonstrated the efficacy of biological therapies in CRSwNP, important clinical questions remain unresolved. These include optimal patient selection, the appropriate timing of biologic initiation in relation to endoscopic sinus surgery, comparative positioning of available biologic agents, identification of predictive biomarkers of response, and strategies for patients with inadequate response or the need for switching therapy. Therefore, this review aims to provide a critical state-of-the-art synthesis of current evidence, focusing not only on efficacy outcomes but also on practical aspects of treatment decision-making and personalized management of severe CRSwNP [11,12,13,14,15,16,17,18].

## 2. Methodology

The present study was conducted as a narrative review with the objective of critically synthesizing the current evidence on the role of biological therapies in the management of CRSwNP. Relevant literature was identified through searches of the electronic databases PubMed, Scopus, and Web of Science. The search strategy was based on a combination of Medical Subject Headings (MeSH) and relevant free-text terms, including: “chronic rhinosinusitis with nasal polyps”, “CRSwNP”, “biological therapy”, “monoclonal antibodies”, “dupilumab”, “omalizumab”, “mepolizumab”, “benralizumab”, and “type 2 inflammation”. The search was limited to articles published in English between 2015 and 2025, in order to ensure the inclusion of up-to-date data reflecting recent advances in immunopathology and targeted therapies, as well as contemporary clinical practice guidelines (including European Position Paper on Rhinosinusitis and Nasal Polyps (EPOS) and European Academy of Allergy and Clinical Immunology/European Forum for Research and Education in Allergy and Airway Diseases (EAACI/EUFOREA) recommendations). The identified literature was selected based on its relevance to the objectives of this review. Priority was given to randomized controlled trials, systematic reviews, meta-analyses, and international clinical guidelines addressing the pathophysiological mechanisms underlying CRSwNP, particularly those related to type 2 inflammation, the mechanisms of action of biological agents, and their clinical efficacy, safety, and positioning within current therapeutic algorithms. Exclusion criteria included case reports, conference abstracts without full-text availability, and non-peer-reviewed publications. Relevant data were narratively synthesized with a focus on key clinical and mechanistic aspects, including therapeutic targets, treatment outcomes (e.g., Nasal Polyp Score, SNOT-22), safety profiles, and eligibility criteria for biological therapy. Due to the heterogeneity of the included studies with respect to design, patient populations, and outcome measures, a quantitative meta-analysis was not appropriate; therefore, the findings are presented as a qualitative, descriptive synthesis of the available evidence.

As this work is a narrative review based exclusively on previously published literature, no formal systematic review methodology was applied, no quality assessment of individual studies was performed, and no PRISMA flow diagram was generated. Consequently, this review should not be interpreted as a comprehensive systematic review but rather as a critical narrative overview of the available evidence.

As this study is based exclusively on previously published data and does not involve direct participation of human subjects or animals, ethical approval was not required. 

### Limitations

This review has several limitations. First, it was conducted as a narrative review rather than a systematic review; therefore, no formal meta-analysis was performed. Although the literature search was designed to identify relevant and up-to-date evidence, the absence of a predefined systematic protocol limits the reproducibility of the search and study-selection process and may have resulted in the omission of some relevant publications. In addition, the available evidence on biological therapies for CRSwNP remains heterogeneous with respect to study design, patient characteristics, outcome measures, and follow-up duration, limiting direct comparisons across studies. Long-term comparative data, particularly head-to-head studies evaluating different biological agents, remain limited. Finally, the availability, regulatory approval, and reimbursement policies for biological therapies vary considerably among countries, which may affect the generalizability and implementation of the therapeutic recommendations discussed in this review.

## 3. Standard Treatment and Its Limitations

### 3.1. Glucocorticosteroids 

Glucocorticosteroids (GCS) constitute the cornerstone of pharmacological management of CRSwNP and—according to both European and American guidelines—are recommended as first-line therapy in patients with pronounced inflammation of the nasal and sinus mucosa. Their widespread use is attributed to the synergistic combination of anti-inflammatory, immunosuppressive, and anti-edematous properties, which enable suppression of the local inflammatory response, reduction in tissue edema, and decrease in nasal polyp volume. Clinically, this translates into improved nasal patency, reduced secretion, and partial or complete restoration of olfaction [19,20]. 

From a mechanistic perspective, GCS administered intranasally or systemically bind to cytoplasmic glucocorticoid receptors, and the resulting complex translocates to the nucleus to regulate gene expression. Their main effect in CRSwNP is the suppression of type 2 inflammatory cytokines, particularly IL-4, IL-5, and IL-13, leading to reduced eosinophilic inflammation and decreased recruitment and activation of eosinophils, basophils, and mast cells. GCS also reduce mucus production and tissue remodeling, contributing to improved mucosal function and disease control. In addition, rapid non-genomic anti-inflammatory effects, such as modulation of intracellular signaling pathways, may further enhance their therapeutic activity [21].

In routine clinical practice, intranasal corticosteroids (INCS) are the most commonly used form. Agents such as mometasone, fluticasone, and budesonide are recommended as baseline therapy for CRSwNP due to their ability to achieve high local drug concentrations with minimal systemic exposure. Numerous studies have demonstrated that regular, long-term use of INCS leads to reduced polyp size, alleviation of nasal obstruction, improved olfaction, and decreased scores on the SNOT-22, reflecting a meaningful improvement in quality of life. A systematic review and network meta-analysis confirmed the significant efficacy of topical GCS in CRSwNP [19,22,23]. 

When topical therapy is insufficient, short courses of systemic corticosteroids (e.g., prednisone or methylprednisolone) provide rapid reduction in inflammation, polyp size, and symptom severity. However, due to the risk of significant adverse effects—including osteoporosis, metabolic disturbances, hypertension, and adrenal suppression—their use should be limited to short courses under medical supervision. Even brief treatment has been associated with an increased risk of systemic complications [24,25].

Recent advances in pharmaceutical technology have led to the development of more sophisticated delivery systems, such as biodegradable, mometasone-eluting implants (e.g., Propel™), used intranasally during endoscopic sinus surgery. These implants enable sustained, localized drug release over approximately 30 days, providing prolonged anti-inflammatory effects at the disease site without significant systemic exposure. Clinical studies have demonstrated that in patients with severe, recurrent CRSwNP and comorbid asthma, such implants reduce recurrence rates, decrease the need for revision surgery, and limit the use of systemic corticosteroids. Nevertheless, further long-term studies in larger patient populations are required [26,27,28]. 

Despite their high efficacy, GCS have certain limitations. Meta-analyses indicate that approximately one-third of patients with CRSwNP do not achieve complete remission despite standard intranasal therapy. In particular, patients with neutrophilic (type 1 or type 3) inflammation exhibit reduced responsiveness to GCS, necessitating individualized treatment approaches. In such cases, biological therapies targeting specific immune pathways, modification of pharmacotherapy regimens, or surgical intervention may be required. Current studies also highlight the importance of assessing SCS burden and its impact on quality of life and treatment outcomes [20]. 

According to the EPOS 2020 recommendations, intranasal corticosteroids and saline irrigations are the cornerstone of long-term CRS management. Short courses of oral corticosteroids may be used in CRSwNP patients with severe inflammation and olfactory dysfunction, while endoscopic sinus surgery is recommended for persistent disease despite appropriate medical therapy to improve sinus ventilation and topical drug delivery. In selected patients with severe uncontrolled type 2 inflammation, biologics may be added to standard treatment. Routine use of antihistamines, antifungals, decongestants, mucolytics, proton-pump inhibitors, and prolonged antibiotics is not recommended unless justified by coexisting conditions or specific clinical indications [29].

Future directions in CRSwNP management include optimization of drug delivery systems, identification of predictive biomarkers of response to GCS, development of combination therapies, and personalization of treatment based on inflammatory endotypes and comorbidities. A protocol for a systematic analysis comparing different dosing regimens and treatment durations has recently been published [30]. 

GCS therapy remains a fundamental component of CRSwNP treatment, enabling effective control of type 2 inflammation, symptom relief, reduction in polyp burden, and improved quality of life. However, a subset of patients requires individualized therapeutic strategies, including biological or surgical approaches [26,28].

### 3.2. Antihistamines 

Antihistamines, which act as H_1_ receptor antagonists, play a limited and primarily adjunctive role in the treatment of CRSwNP. Their mechanism involves blocking the H_1_ receptor, thereby inhibiting the effects of histamine—a key mediator of type I allergic reactions responsible for symptoms such as itching, sneezing, and rhinorrhea. However, the dominant process in CRSwNP is chronic type 2 inflammation driven by eosinophils, Th2 lymphocytes, and cytokines such as IL-4, IL-5, and IL-13. Histamine plays a secondary role; therefore, H_1_ receptor blockade does not significantly affect the underlying immunological mechanisms [25]. 

According to current clinical guidelines, antihistamines are not recommended as primary therapy for CRSwNP. Their use is justified mainly in patients with coexisting allergic rhinitis. While they may alleviate allergic symptoms and improve patient comfort, they do not affect polyp size or mucosal inflammation [31,32,33]. 

Second-generation oral antihistamines, such as cetirizine, loratadine, and fexofenadine, have favorable safety profiles and effectively control allergic symptoms. In CRSwNP, they function as supportive therapy, providing moderate improvement in nasal symptoms without affecting objective measures such as NPS or SNOT-22 [25]. 

Intranasal antihistamines, particularly azelastine, may offer additional benefits due to anti-inflammatory and mast cell-stabilizing properties. Studies have shown reductions in inflammatory markers such as eosinophil cationic protein (ECP) and myeloperoxidase (MPO), suggesting potential utility in patients with an allergic component [34]. 

Antihistamines are generally well tolerated, with minimal adverse effects. Intranasal azelastine may cause a transient bitter taste or mild drowsiness, but long-term data indicate no significant systemic risks [31,35]. 

EPOS 2020 does not recommend antihistamines as routine treatment for chronic rhinosinusitis with nasal polyps because available evidence does not demonstrate a meaningful effect on the underlying inflammatory process or disease control. Their use should be limited to patients with concomitant allergic rhinitis or other IgE-mediated allergic conditions, in whom they may improve symptoms such as sneezing, itching, and rhinorrhea. The guidelines further classify antihistamines among therapies with limited evidence or no strong recommendation for CRSwNP treatment itself [36]. 

In summary, antihistamines do not constitute causal therapy for CRSwNP and should be considered only as adjunctive treatment in patients with concomitant allergic conditions [31,37]. 

### 3.3. Surgical Treatment

Functional endoscopic sinus surgery (FESS) is an established strategy used in patients with chronic sinusitis in whom pharmacological treatment has failed. FESS is a set of minimally invasive techniques aimed at directly opening the air cells of the sinuses in order to restore proper function and ventilation of the sinuses [29].

FESS is mainly used in the treatment of CRS, especially in patients in whom pharmacological treatment has not been effective. CRS may have various etiologies, which makes conservative treatment less effective; therefore, patients resistant to therapy are qualified for surgery. Another important indication for FESS is nasal polyps, which can impair pharmacological treatment and obstruct airflow in the nasal cavity, often requiring surgical intervention.

In patients with CRSwNP refractory to conservative treatment, FESS significantly improved nasal obstruction and clinical symptoms, confirming its effectiveness after failure of medical therapy [38]. 

One study evaluated the recurrence rate of nasal polyps in patients with CRSwNP after FESS in a multicenter cohort. Despite surgery and continued medical therapy, approximately 40% of patients experienced polyp recurrence after 18 months of follow-up, which is comparable to other studies. Significant risk factors for recurrence included previous FESS and greater severity of polyposis before surgery. It was shown that FESS improves symptom control in most patients, but endoscopic disease control remains limited, and polyp recurrence still affects a significant proportion of patients [39]. FESS has been shown to be significantly more effective in reducing symptoms compared to long-term low-dose clarithromycin and topical therapy. The findings suggest that FESS should be considered in patients in whom topical therapy alone does not provide adequate symptom control [40].

In recent years, the surgical approach in CRSwNP has changed due to the development of endotype-based classification and treatment strategies. However, no consensus has yet been reached regarding the optimal surgical strategy.

## 4. Biological Therapies: Mechanism, Types, and Effectiveness

### 4.1. Inclusion Criteria

Biologic therapy in chronic rhinosinusitis with nasal polyps is reserved for patients with a severe, uncontrolled, diffuse type 2 inflammatory endotype who continue to exhibit a substantial residual disease burden despite appropriate intranasal corticosteroids and, in most cases, endoscopic sinus surgery. EPOS 2020, as summarized in later consensus documents, defines “difficult to treat” CRSwNP as disease in which acceptable control is not achieved despite appropriate endoscopic sinus surgery, continuous INCS, and up to two short (7–21 day) courses of SCS and/or long-term (>3 months) low-dose steroids in the previous year [41]. EPOS 2020 and the subsequent EPOS/EUFOREA 2023 update converge on a structured, criteria-based approach to patient selection centered on markers of type 2 inflammation, systemic corticosteroid exposure, health-related quality of life, olfactory function, and lower airway comorbidity. In patients with bilateral CRSwNP who have undergone ESS, biologics are indicated when at least three of the following five criteria are fulfilled: (1) objective evidence of type 2 inflammation, originally defined in EPOS 2020 by blood eosinophils ≥250 cells/μL or tissue eosinophils ≥10 per high-power field or total IgE ≥ 100 kU/L, with the 2023 EPOS/EUFOREA update lowering the blood eosinophil threshold to ≥150 cells/μL while maintaining eosinophils as the dominant biomarker of type 2 endotype; (2) a need for repeated systemic corticosteroid courses (typically ≥2 per year) or a contraindication to systemic corticosteroids; (3) significantly impaired disease-specific quality of life, commonly operationalized as a Sinonasal Outcome Test-22 (SNOT-22) score ≥ 40; (4) significant loss of smell, i.e., anosmia on objective olfactory testing; and (5) comorbid asthma, reflecting unified type 2 airway inflammation. For non-surgical patients, a more stringent threshold is recommended, with fulfillment of at least four of these five criteria proposed before initiation of biologic therapy [11,12,13]. EAACI/EUFOREA consensus further emphasizes that severe CRSwNP eligible for biologics is typically associated with type 2-high biomarkers and comorbidities such as asthma, NSAID-exacerbated respiratory disease (N-ERD), and allergy, which both support the type 2 endotype and may themselves benefit from systemic biological therapy.

### 4.2. Available Biological Therapies

Refractory chronic rhinosinusitis is a condition predominantly driven by type 2 inflammation mediated by IL-4, IL-5, IL-13, and IgE [10,42,43]. In patients who remain uncontrolled despite INCS, SCS, and FESS, biologics provide targeted modulation of these pathways through monoclonal antibodies directed against IL-4/IL-13, IL-5/IL-5R, or IgE [44,45,46]. By selectively inhibiting key inflammatory mediators, these agents reduce polyp burden, improve symptoms and quality of life, and decrease the need for SCS and revision surgery, thereby addressing the underlying endotype rather than only symptomatic manifestations [47,48,49]. Biological treatment includes monoclonal antibodies such as dupilumab (anti-IL-4Rα), omalizumab (anti-IgE), mepolizumab, and benralizumab (anti-IL-5/IL-5R) [48]. Clinical trial data suggest that biologics may be a beneficial option in patients with treatment-refractory CRSwNP, particularly in those who have not achieved disease control despite topical GCS or after sinus surgery. Biologics are mainly considered in patients with severe disease, frequent relapses, need for repeated steroid courses, or failure of surgical treatment, and their use aims to control type 2 inflammation and reduce symptoms and recurrence of nasal polyps [50].

Dupilumab is a fully human IgG4 monoclonal antibody that targets the interleukin-4 receptor alpha subunit (IL-4Rα), the shared component of type I (IL-4Rα/γc) and type II (IL-4Rα/IL-13Rα1) receptor complexes for IL-4 and IL-13, thereby imposing a dual blockade of IL-4– and IL-13–mediated signaling [51,52,53]. By binding IL-4Rα, dupilumab prevents ligand-induced receptor assembly and downstream activation of type 2-associated pathways in multiple structural and immune cells relevant to CRSwNP, including epithelial cells, goblet cells, fibroblasts, eosinophils, and B cells [51,52,53]. In CRSwNP, where IL-4 and IL-13 are key upstream cytokines that initiate and perpetuate type 2 inflammation and tissue remodeling, IL-4Rα inhibition with dupilumab interrupts processes such as IgE class switching, eosinophil recruitment, goblet cell hyperplasia, mucus hypersecretion, and profibrotic remodeling [54,55]. Dupilumab is a key drug used in type 2 inflammatory diseases such as asthma and atopic dermatitis. It was the first biologic approved by Italian Medicines Agency (AIFA) in 2020 for adults with severe CRSwNP, as an add-on to INCS in patients refractory to medical and/or surgical treatment [56]. Biomarker analyses in patients with CRSwNP demonstrate that dupilumab reduces concentrations of multiple type 2 inflammatory mediators in nasal secretions and blood, and downregulates local IgE production and eosinophil homing, consistent with broad suppression of the IL-4/IL-13 axis [10]. Through this targeted interference with a central cytokine hub, dupilumab directly addresses the type 2 endotype in CRSwNP and underpins its clinical efficacy as the first IL-4/IL-13–pathway–directed biologic approved for uncontrolled disease [51,52,54].

Omalizumab is a humanized IgG1 monoclonal antibody that specifically targets IgE, a pivotal driver of type 2 inflammation in CRSwNP. By binding to free IgE at the Cε3 domain, omalizumab sterically hinders its interaction with high- and low-affinity IgE receptors (FcεRI, FcεRII) on mast cells, basophils, and antigen-presenting cells, thereby preventing receptor cross-linking and subsequent cell activation, degranulation, and release of pro-inflammatory mediators [57,58,59]. Omalizumab also accelerates dissociation of IgE from FcεRI and induces a rapid downregulation of FcεRI expression on effector and antigen-presenting cells, further dampening IgE-dependent signaling [57,58]. In CRSwNP, where locally produced, polyclonal, functionally active IgE in polyp tissue sustains eosinophilic, T2-high inflammation independent of systemic atopy, this reduction in free and receptor-bound IgE disrupts the local inflammatory cascade, limiting recruitment and activation of eosinophils and other type 2 effector cells [60,61,62]. Clinically, this immunomodulation translates into decreased nasal polyp size, improved nasal obstruction and olfactory function, and reduced symptom burden and need for SCS or surgery, as demonstrated in randomized phase III trials and real-life cohorts [60,61,62].

Mepolizumab is a humanized monoclonal IgG1 antibody that selectively targets IL-5, a key type 2 cytokine that drives eosinophil differentiation, recruitment, activation, and survival in CRSwNP [63,64,65]. By binding IL-5 with high affinity, mepolizumab prevents its interaction with IL-5 receptor alpha on eosinophils, thereby selectively inhibiting IL-5–dependent eosinophilic inflammation without direct interference with other cytokines [63,64,66]. In type 2 CRSwNP, which is characterized by eosinophilic tissue infiltration and elevated IL-5 levels, this blockade leads to marked reductions in blood and tissue eosinophil counts and in eosinophil-associated mediators, including ECP and other local markers of type 2 inflammation [63,64,66]. Histological and tissue studies show that mepolizumab decreases inflammatory eosinophil subpopulations within nasal polyp tissue and can be associated with restoration of mucosal architecture, including reduced edema and goblet cell hyperplasia [66,67]. Clinically, these immunologic effects translate into reduced nasal polyp size, improved nasal obstruction, better sinonasal symptom scores, and decreased need for SCS and sinus surgery in patients with severe, refractory, often eosinophilic CRSwNP [63,64,68,69]. Thus, mepolizumab exerts its therapeutic role in type 2 CRSwNP by selectively neutralizing IL-5, attenuating eosinophil-driven inflammation central to polyp pathogenesis and recurrence.

Benralizumab is a humanized, afucosylated IgG monoclonal antibody that selectively targets the interleukin-5 receptor alpha subunit (IL-5Rα), which is highly expressed on eosinophils and, to a lesser extent, basophils [70,71,72]. By binding IL-5Rα, benralizumab not only blocks IL-5 signaling but, critically, engages FcγRIIIa (CD16a) on natural killer (NK) cells, triggering antibody-dependent cell-mediated cytotoxicity (ADCC) and rapid, near-complete depletion of circulating and tissue eosinophils through apoptosis [70,71,72]. Experimental work demonstrates caspase-3/7 activation, cytochrome c upregulation, and eosinophil death in autologous co-culture systems, with additional contributions from macrophage-mediated antibody-dependent cellular phagocytosis and TNF-dependent amplification of apoptosis [73]. In CRSwNP, eosinophilic CRS and nasal polyps are characterized by intense IL-5–driven eosinophilic infiltration of polyp tissue, elevated eosinophil-derived mediators, and concordance between blood and tissue eosinophilia. Clinical studies in eosinophilic CRSwNP and in severe eosinophilic asthma with comorbid nasal polyps show that benralizumab-induced eosinophil depletion (often to 0 cells/µL in blood and to nearly 0 in polyp tissue) is accompanied by reductions in NPS, sinus opacification, and type 2 inflammatory markers, alongside improvements in symptom scores and quality of life, particularly in highly eosinophilic phenotypes [70,74,75]. Thus, through IL-5Rα-directed ADCC-mediated eosinophil apoptosis, benralizumab directly targets the eosinophilic component of type 2 inflammation that sustains polyp growth and persistence in eosinophilic nasal polyps. Anti-IL-5 biologics are indicated in systemic eosinophilic disorders, especially EGPA, because IL-5 is a master regulator of eosinophil maturation, survival, and tissue trafficking, and EGPA is defined by eosinophilic inflammation in blood and tissues that damages both the respiratory tract and small-to-medium vessels [76,77,78]. Mepolizumab neutralizes IL-5, whereas benralizumab targets IL-5Rα and additionally induces eosinophil apoptosis through antibody-dependent cell-mediated cytotoxicity, which explains the particularly profound depletion of circulating and tissue eosinophils seen with benralizumab [79]. This mechanism is relevant to severe type 2 CRSwNP because IL-5-rich sinonasal disease is characterized by tissue eosinophilia, local IL-5 expression, and eosinophil-driven remodeling that promotes persistent polyp formation rather than a purely localized mucosal process [80,81]. Severe CRSwNP and EGPA are therefore immunologically linked by a shared systemic type 2 eosinophilic endotype in which eosinophils infiltrate airway tissues, sustain inflammation, and contribute to organ damage across the united airway and beyond [82,83,84]. In EGPA, that same eosinophil program extends outside the sinonasal mucosa into extravascular tissues and vessel walls, producing the dual phenotype of refractory sinonasal polyposis and eosinophil-associated vasculitic or granulomatous disease [77,85].

Tezepelumab is a human monoclonal antibody directed against thymic stromal lymphopoietin (TSLP), an epithelial alarmin positioned at the top of the sinonasal inflammatory cascade in CRSwNP, and it exerts its effect by binding soluble TSLP and preventing its engagement with the heteromeric TSLP receptor complex composed of TSLPR and IL-7Rα [86,87]. By blocking this proximal epithelial danger signal rather than a single downstream effector pathway, tezepelumab functions as an upstream barrier-level immunomodulator capable of intercepting inflammation before amplification through canonical type 2 cytokine circuits [88,89]. This mechanism is especially relevant in CRSwNP because TSLP expression is increased in diseased sinonasal mucosa, is higher in eosinophilic than non-eosinophilic CRSwNP, is predominantly localized to epithelial cells, and correlates with both symptom burden and tissue type 2 cytokine expression [88,90]. TSLP is released by barrier epithelium in response to environmental and mechanical injury signals and serves as a frontline alarmin that conditions dendritic cells, innate lymphoid cells, and other immune effectors to initiate and sustain allergic airway inflammation [91,92,93]. In CRSwNP specifically, TSLP and its functional cleavage products activate myeloid dendritic cells and ILC2s, and tezepelumab dose-dependently inhibits both full-length and post-translationally truncated TSLP species present in nasal polyp and ethmoid tissue, thereby suppressing mDC-associated chemokine output such as CCL17 and CCL22 and ILC2-derived cytokine production such as IL-5 and IL-13 [94]. Because TSLP regulates the differentiation and activation of ILC2 and Th2 pathways and is a potent inducer of type 2 immunity, its neutralization blunts the downstream elaboration of the canonical effector cytokines IL-4, IL-5, and IL-13, with clinical asthma datasets consistently showing suppression of eosinophils, FeNO, IL-5, IL-13, and IgE after TSLP blockade [93,95,96]. This downstream dampening is mechanistically important for polyp disease because alarmin-activated ILC2s drive IL-5- and IL-13-mediated eosinophilic inflammation, goblet cell hyperplasia, mucus hypersecretion, and tissue remodeling, all of which are central to nasal polyp growth and persistence [92,97]. Tezepelumab also appears to disrupt feed-forward epithelial inflammatory loops, since TSLP participates in reciprocal amplification with IL-33 and its receptors in human nasal epithelial cells, a network that facilitates persistent Th2-skewed inflammation in eosinophilic CRSwNP [90]. The biologic plausibility of this strategy is reinforced by evidence that anti-TSLP therapy acts more proximally than blockade of IgE, IL-5, or IL-4/IL-13, and therefore may suppress broader inflammatory programs, including eosinophilic recruitment, mucosal edema, mucus overproduction, and remodeling processes that culminate in polypogenesis [89,98,99]. Taken together, tezepelumab is best framed in severe CRSwNP as a barrier-level immunomodulator that neutralizes the epithelial alarmin TSLP upstream of downstream effector cytokines, suppresses mDC and ILC2 activation, and is therefore biologically rational for uncontrolled, alarmin-dominated type 2-high CRSwNP phenotypes [96,100].

Dupilumab, omalizumab, and mepolizumab are formally approved by both the FDA and EMA as add-on maintenance treatments for severe, uncontrolled CRSwNP, and the current review literature consistently identifies these three agents as the only established CRSwNP approvals across the United States and Europe [45,69,101]. Dupilumab was the first FDA-approved biologic for CRSwNP in 2019 and subsequently received EMA approval for severe CRSwNP, while omalizumab received CRSwNP approval in 2020 and mepolizumab in 2021, with recent region-specific reviews also confirming ongoing adult CRSwNP approvals in Europe and other markets including Italy, Spain, and China [45,102,103]. Benralizumab, despite phase 3 evaluation in CRSwNP, real-world use, and continued inclusion in comparative efficacy analyses, remains unapproved for standalone CRSwNP and is therefore off-label or investigational in this indication, whereas its recognized licensed uses are in severe eosinophilic asthma and EGPA rather than nasal polyposis [104,105,106]. Tezepelumab occupied an investigational position for CRSwNP through 2025, when reviews still described it as being in phase 3 development [45,107]; however, by early 2026 US otolaryngology review literature characterized it as a fourth approved biologic for CRSwNP in the United States [108], and contemporaneous post-WAYPOINT comparative analyses treated phase 3 tezepelumab data as demonstrating significant CRSwNP benefit comparable overall to dupilumab, with favorable hazard-ratio signals for oral corticosteroid use and/or surgery [99,109]. Accordingly, as of 2026, tezepelumab appears to be positioned as FDA-approved in the United States for severe CRSwNP after WAYPOINT, while equivalent EMA approval is not established in the supplied evidence and its status outside the US remains regionally heterogeneous or still emerging [101,107,108].

### 4.3. Clinical Efficacy

In adults with severe, refractory CRSwNP, pivotal phase 3 randomized controlled trials demonstrate that biological therapies targeting type 2 inflammation yield significant and clinically meaningful improvements across objective and patient-reported outcomes. In LIBERTY NP SINUS-24 and SINUS-52, dupilumab plus INCS produced robust reductions in polyp size, nasal obstruction, and sinus opacification at week 24, with least-squares mean differences versus placebo in NPS of −2.06 (95% CI −2.43 to −1.69; *p* < 0.0001) and −1.80 (−2.10 to −1.51; *p* < 0.0001), nasal congestion of −0.89 (−1.07 to −0.71; *p* < 0.0001) and −0.87 (−1.03 to −0.71; *p* < 0.0001), and Lund–Mackay CT scores of −7.44 (−8.35 to −6.53; *p* < 0.0001) and −5.13 (−5.80 to −4.46; *p* < 0.0001) in SINUS-24 and SINUS-52, respectively [110]. These benefits emerged rapidly and were sustained through 52 weeks in the continuous-dupilumab arms, alongside marked improvements in smell and health-related quality of life, with clinically relevant reductions in need for SCS and repeat surgery reported [110]. In the replicate POLYP 1 and POLYP 2 trials, omalizumab significantly improved both co-primary endpoints at week 24: mean changes in NPS were −1.08 versus 0.06 (*p* < 0.0001) and −0.90 versus −0.31 (*p* = 0.0140), and nasal congestion scores improved by −0.89 versus −0.35 (*p* = 0.0004) and −0.70 versus −0.20 (*p* = 0.0017) versus placebo, respectively [61]. Omalizumab also yielded large SNOT-22 reductions, −24.7 versus −8.6 and −21.6 versus −6.6 (both *p* < 0.0001), exceeding the minimal clinically important difference and indicating substantial quality-of-life gains, with significant improvements in University of Pennsylvania Smell Identification Test (UPSIT), sense of smell, postnasal drip, and rhinorrhea confirming broad symptomatic benefit [61]. An international, multicentre, randomized, double-blind phase 4 trial EVEREST established clinically important comparative evidence for biologic selection in type 2-high CRSwNP with asthma by testing dupilumab directly against omalizumab in the first head-to-head respiratory biologic trial rather than against placebo alone. The study contained adults with severe uncontrolled CRSwNP, persistent nasal congestion and loss of smell, physician-diagnosed asthma, and background mometasone therapy, then assigning 360 patients 1:1 to dupilumab or omalizumab for 24 weeks The central efficacy result was that dupilumab was superior to omalizumab across all primary and secondary endpoints at week 24, including a greater reduction in endoscopic Nasal Polyp Score, with more effective regression of polyp burden. Dupilumab also achieved broader symptom advantages across secondary efficacy endpoints [111]. The SYNAPSE trial showed that mepolizumab added to standard of care significantly reduced polyp size at week 52 (adjusted difference in medians for endoscopic NPS −0.73, 95% CI −1.11 to −0.34; *p* < 0.0001) and improved mean nasal obstruction Visual Analog Scale (VAS) during weeks 49–52 by −3.14 (95% CI −4.09 to −2.18; *p* < 0.0001) versus placebo, representing a large and clinically important improvement in nasal patency [64]. Mepolizumab significantly decreased the proportion of patients requiring nasal surgery and systemic corticosteroids, improved loss-of-smell VAS and peak nasal inspiratory flow, and produced SNOT-22 reductions almost twice the 8.9-point minimal clinically important difference, with effects evident by week 4 and sustained through week 52 [64]. In OSTRO, benralizumab met both co-primary endpoints at week 40, significantly improving NPS and nasal blockage scores versus placebo (*p* ≤ 0.005) [71]. Key secondary analyses showed nominally significant improvements in difficulty with sense of smell at weeks 40 and 56 (*p* = 0.003 and *p* = 0.002) and in SNOT-22 at week 56 (*p* = 0.02), although improvements in SNOT-22 at week 40 and in time to first surgery and/or systemic corticosteroid use did not reach statistical significance, with trends favoring benralizumab (e.g., *p* = 0.07 for surgery/systemic corticosteroids) [70,71]. In the phase 3 WAYPOINT trial, tezepelumab demonstrated robust clinical efficacy in adults with severe, uncontrolled CRSwNP, with 203 patients assigned to tezepelumab and 205 to placebo over 52 weeks [86,112]. Tezepelumab significantly reduced total Nasal Polyp Score versus placebo at week 52, with a mean difference of −2.07 (95% CI, −2.39 to −1.74), and also improved mean nasal congestion score by −1.03 (95% CI, −1.20 to −0.86), confirming benefit across both objective endoscopic burden and cardinal symptom severity [86,96,112]. Health-related quality of life improved substantially, as reflected by a SNOT-22 mean difference of −27.26 versus placebo, alongside significant gains in loss-of-smell score, Lund–Mackay score, and total symptom score, indicating broad disease control beyond polyp size alone [86,112]. Tezepelumab also reduced the requirement for rescue treatment, with systemic glucocorticoid use occurring in 5.2% of treated patients versus 18.3% with placebo (hazard ratio 0.12, 95% CI 0.04–0.27), while the need for surgery was likewise markedly lower, supporting its relevance in difficult-to-treat disease [86,99,112]. Taken together, recent comparative appraisals place tezepelumab among the highest-performing biologics for reduction in polyp burden, relief of congestion, and reduction in corticosteroid or surgical rescue, supporting its emergence as a highly active upstream therapeutic strategy in refractory type 2-high CRSwNP [86,96]. 

Benralizumab was noninferior to mepolizumab for remission in the phase 3 head-to-head MANDARA trial, with adjusted remission rates at weeks 36 and 48 of 59% versus 56%, respectively, while accrued remission duration and time to first relapse were similar between groups [76]. Oral corticosteroid sparing favored benralizumab, with complete withdrawal during weeks 48 through 52 achieved in 41% of patients compared with 26% with mepolizumab [76,113]. More stringent post hoc analyses further showed that remission off oral glucocorticoids was achieved more often with benralizumab than with mepolizumab, supporting the clinical relevance of steroid-free disease control [114]. Sustained 100% oral glucocorticoid reduction was maintained by 24.3% of benralizumab-treated patients versus 10.0% of mepolizumab-treated patients, consistent with a greater capacity for complete steroid withdrawal over time [113]. Together, these data support deep eosinophil depletion through IL-5Rα targeting as a mechanistically plausible strategy in overlapping upper airway and systemic eosinophilic disease, where durable remission and corticosteroid minimization are shared therapeutic goals [76,113]. 

In a multicenter Canadian study, use and switching of type 2 biologics were analyzed. The most common scenario was switching from mepolizumab to dupilumab, mainly due to insufficient sinus symptom control. The study showed that switching between biologics is not always effective, and switching within anti-IL-5 agents rarely leads to significant improvement, while switching to a drug with a different mechanism of action is more effective. In cases of failure of one biologic, particularly anti-IL-5, switching to a different target agent such as dupilumab may be more beneficial [14]. Beyond biologic monotherapy or strategic drug switching, emerging clinical evidence addresses the utility of combination biologic therapy in complex, overlapping phenotypes. Severe, refractory CRSwNP that overlaps with EGPA or related systemic eosinophilic disease provides a biologically plausible setting for dual-targeting because anti-IL-5/IL-5R therapy suppresses eosinophil-dependent systemic inflammation and steroid burden, whereas dupilumab more effectively improves rhinologic endpoints linked to local type 2 tissue remodeling and olfactory dysfunction [115,116]. This complementarity is reinforced by phenotype-level observations that anti-IL-5 agents are favored in eosinophilic or systemic-manifestation-dominant disease, while dupilumab shows stronger benefit for sinonasal-dominant CRSwNP, particularly for nasal symptoms, polyp burden, and smell outcomes [101,117]. Mechanistically, mepolizumab neutralizes IL-5 and benralizumab targets IL-5Rα to achieve rapid, near-complete eosinophil depletion, thereby addressing the eosinophilic vasculitic axis that sustains EGPA and severe eosinophilic asthma, while dupilumab blocks IL-4Rα and inhibits IL-4/IL-13 signaling, a pathway more closely linked to epithelial inflammation, mucus production, and remodeling in CRSwNP [101,103,118]. Emerging clinical evidence remains early but is directionally consistent: in two EGPA cases with systemic remission maintained on anti-IL-5/IL-5R therapy, add-on dupilumab produced substantial reductions in SNOT-22 and nasal polyp scores while BVAS remained 0, directly supporting compartment-specific rescue of refractory sinonasal disease without loss of vasculitis control [119]. A separate relapsing-refractory MPO-ANCA-positive EGPA case that developed marked eosinophilia and worsening asthma after switching to dupilumab alone improved after dupilumab-benralizumab combination, with better nasal and ear outcomes, improved asthma control, reduced blood eosinophils, and no adverse effects over 12 months, suggesting that IL-5R blockade can also buffer dupilumab-associated hypereosinophilia in selected patients [120]. Broader real-world combination-biologic cohorts, although not CRSwNP-specific, support feasibility and short- to mid-term tolerability, with 25 patients receiving concurrent anti-type 2 biologics for a median of 17.5 months showing no anaphylaxis, immune dysfunction, pneumonia, or malignancy, and pooled literature-level experience across 51 reported combinations showing only one mild adverse reaction overall [120,121]. This strategy remains supported mainly by case-based and retrospective evidence rather than controlled trials, and current reviews still emphasize the need for endotype-driven selection, multidisciplinary management, and prospective validation; however, the available data support combination anti-IL-5/IL-5R plus anti-IL-4/IL-13 therapy as a rational, apparently well-tolerated, and potentially synergistic approach for selected patients with alarmin-rich, tissue-remodeling CRSwNP superimposed on systemic eosinophilic disease in whom monotherapy controls only one compartment of inflammation [46,118,119].

### 4.4. Safety

Mepolizumab, benralizumab, omalizumab, and dupilumab show generally favourable safety and tolerability profiles in phase 3 CRSwNP trials, with overall adverse event rates similar to placebo and few treatment-related serious events. In SYNAPSE, on-treatment adverse events (AEs) occurred in 82% with mepolizumab versus 84% with placebo, with nasopharyngitis, headache, epistaxis, and sinusitis the most frequent events; investigator-judged treatment-related AEs were modestly higher with mepolizumab (15% vs. 9%), while serious AEs were identical (6% vs. 6%) and none were considered treatment-related, with no mepolizumab-treated deaths and low-titer, non-neutralising anti-drug antibodies in a small minority of patients [64]. In EVEREST safety was broadly similar over 24 weeks, with treatment-emergent adverse events reported in 64% of dupilumab-treated and 67% of omalizumab-treated participants and no deaths, so the efficacy separation was not offset by a major short-term safety penalty in the available data [111]. In OSTRO, 77.3% of benralizumab-treated and 78.8% of placebo-treated patients had ≥1 AE, predominantly mild–moderate, with nasopharyngitis, asthma, headache, and viral upper respiratory tract infection most common and not increased versus placebo; serious AEs and discontinuations due to AEs were similar between groups, and no deaths occurred [71]. In POLYP 1/2, omalizumab was “well tolerated”, with an AE profile (headache, injection-site reactions, arthralgia, dizziness, upper abdominal pain) consistent with prior indications and no new safety signals; anaphylaxis, a known rare risk in asthma, was not observed in these CRSwNP trials [61]. In SINUS-24/52, dupilumab’s most frequent AEs (nasopharyngitis, worsening nasal polyps or asthma, headache, epistaxis, injection-site erythema) occurred more often with placebo, and treatment was described as well tolerated without new safety concerns [110]. Across agents, serious treatment-related events are rare, long-term safety up to 52 weeks appears acceptable [64,71,110], and recommended monitoring focuses on routine clinical assessment for injection-site reactions, hypersensitivity, infection, and disease control, with periodic laboratory and immunogenicity monitoring as implemented in the pivotal trials [64,71].

Biological therapy in CRS patients is also associated with a significant reduction in acute disease exacerbations, indirectly assessed by the number of prescribed antibiotics and SCS. A retrospective analysis showed that after initiation of biological therapy, the estimated annual number of antibiotic courses decreased from 1.34 to 0.68, a reduction of approximately 49%. In patients with frequent exacerbations, the reduction was even greater at 62%. A decrease in systemic corticosteroid use of about 60% was also observed. These findings indicate that blocking type 2 inflammatory pathways may reduce the frequency of CRS exacerbations, thereby decreasing antibiotic and steroid use in this patient population [122]. A detailed comparison of granular, agent-specific clinical safety considerations and distinctive post-marketing profiles is presented in Table 1. 

## 5. Discussion

The introduction of biological therapies into the treatment of CRSwNP represents a significant advancement in the therapeutic management of this condition [42,46]. Conventional treatment approaches, including intranasal and SCS as well as surgical interventions, despite their established role, often fail to ensure long-term disease control and are associated with high recurrence rates [42,46]. This limitation is particularly evident in patients with predominant type 2 inflammation, where standard therapies show reduced effectiveness, thereby justifying the need for more targeted treatment strategies [15,42,43].

Biological therapies, based on monoclonal antibodies, enable the selective inhibition of key mediators of type 2 inflammation, including interleukins IL-4, IL-5, IL-13, and immunoglobulin E [42,43,47]. Their mechanism of action directly targets the underlying pathophysiology of CRSwNP, distinguishing them from non-specific anti-inflammatory treatments [16,42,43]. Clinical trials have demonstrated that agents such as dupilumab, omalizumab, mepolizumab, and benralizumab significantly reduce nasal polyp size (as measured by NPS), improve nasal airflow, restore olfactory function, and enhance quality of life (SNOT-22) [12,43,45].

Evidence from the SINUS-24 and SINUS-52 trials (dupilumab), as well as POLYP I/II (omalizumab), confirms significant improvements in NPS and SNOT-22 scores, olfactory test outcomes, and a reduced need for surgery and systemic corticosteroids, even in patients with prior surgical history [12,17,18]. Additionally, their beneficial impact on coexisting eosinophilic asthma and other type 2 inflammatory diseases further reinforces their clinical relevance [18,42,46]. These benefits are also clinically relevant in eosinophilic granulomatosis with polyangiitis (EGPA), a systemic eosinophilic vasculitis in which CRSwNP and severe asthma frequently coexist [133]. 

In particular, anti-IL-5 therapies such as mepolizumab and benralizumab have demonstrated efficacy in controlling eosinophilic inflammation and reducing corticosteroid requirements in EGPA, highlighting the shared pathogenic mechanisms across eosinophilic diseases [76,134].

However, the high cost of biological therapies limits their widespread accessibility, necessitating careful patient selection in accordance with guidelines such as EPOS 2020, updated EPOS/EUFOREA 2023 criteria, and recommendations from EAACI and other expert groups. These criteria take into account disease severity, evidence of type 2 inflammation, history of surgery, and the presence of comorbidities such as asthma [12,17,18]. In patients with concomitant EGPA or other eosinophilic disorders, biologic selection should also consider the potential to control systemic manifestations in addition to sinonasal disease [135].

Treatment response remains variable, and no definitive predictive biomarkers have yet been established. Potential indicators under investigation include blood eosinophil count, serum IgE levels, and type 2 cytokine profiles [15,42,43].

Data from both randomised clinical trials and real-world studies indicate a favourable safety profile, with most adverse events being mild, such as injection-site reactions or upper respiratory symptoms. Nevertheless, long-term safety data remain limited and warrant further investigation [15,17,47].

Currently, biological therapies are primarily recommended for patients with severe, treatment-resistant CRSwNP and recurrent disease following surgery, particularly in the presence of type 2 inflammation [12,17,46]. These therapies significantly improve quality of life, sleep, and olfactory function, while also reducing the need for SCS [15,42,43,46]. As indications for these agents continue to expand, their use in EGPA and other eosinophilic disorders further supports a precision medicine approach targeting common type 2 inflammatory pathways across multiple diseases [136]. 

Future studies should clarify the optimal selection of biologics for patients with overlapping eosinophilic conditions and define biomarkers capable of predicting treatment response across these disease spectra [137].

## 6. Conclusions

This review demonstrates that biologic therapies have substantially changed the treatment paradigm for CRSwNP, particularly in patients with severe, uncontrolled disease despite intranasal corticosteroids, systemic corticosteroids, and/or previous endoscopic sinus surgery. The strongest evidence supports the use of dupilumab, which consistently provides the greatest improvements in nasal polyp size, nasal obstruction, olfactory function, quality of life, and reduces the need for systemic corticosteroids and revision surgery. Omalizumab and mepolizumab have also demonstrated clinically meaningful benefits, particularly in carefully selected patients with type 2 inflammatory disease, although the magnitude of response appears to be less consistent across outcomes.

Current evidence suggests that patients with severe type 2 inflammation, especially those with coexisting asthma, elevated eosinophil counts, or increased IgE levels, derive the greatest benefit from biologic treatment. However, important questions remain regarding the optimal selection of biologic agents, the identification of reliable predictive biomarkers, treatment duration, long-term safety, relapse after treatment discontinuation, and cost-effectiveness in routine clinical practice.

Although intranasal corticosteroids remain the foundation of CRSwNP management and endoscopic sinus surgery continues to play an essential role in patients with persistent disease, biologics represent an effective therapeutic option for appropriately selected patients with refractory CRSwNP. Future studies should focus on direct head-to-head comparisons between biologic agents, long-term real-world outcomes, biomarker-guided patient stratification, and strategies integrating biologic therapy with surgery to establish personalized treatment algorithms.

## Figures and Tables

**Table 1:** Agent-specific safety.

Agent	Common Safety Notes	Distinctive Signals	Injection-Site Reactions
Dupilumab	Usually well tolerated in trials; common events include nasopharyngitis, headache, epistaxis, conjunctivitis, arthralgia, and cough [123,124]	Most distinctive post-marketing profile: ocular events, arthralgia/rheumatic events, rash, and transient eosinophilia; late cutaneous immune-shift events have also been reported [125,126,127]	Higher signal than placebo in NMA; real-world CRSwNP rate 12.7%, higher than ~6% in LIBERTY trials; usually mild local reactions [123,128,129]
Omalizumab	Generally favorable tolerability; common events include headache, nasopharyngitis, and upper respiratory tract symptoms [111,130]	Main differentiator is anaphylaxis signal in comparative pharmacovigilance; pregnancy-related reporting signal also noted in WHO-VigiAccess [105,125]	Reported in about 5.2% of trial participants; typically non-serious [130]
Mepolizumab	Acceptable overall tolerability; headache and fatigue are the most frequent real-world adverse events [17,126]	Safety concerns are less phenotypically distinctive than with dupilumab; broader literature notes rare anaphylaxis, autoantibodies, and herpes zoster [107,123]	Present but not a dominant signal; dose-based NMA suggests higher ISR risk with 100 mg q4w than 75 mg q4w [128,130]
Benralizumab	Low AE-related discontinuation in CRSwNP trials; no significant overall AE excess versus placebo in indirect comparisons [105,131]	No dominant CRSwNP-specific organ-toxicity signal has emerged; current evidence base is smaller than for dupilumab or omalizumab [105,131]	Dose-based NMA suggests somewhat higher ISR risk with 30 mg q4–8w than 20 mg q4–8w [128]
Tezepelumab	CRSwNP safety dataset is still emerging; indirect comparisons have not identified a major overall AE penalty [132]	Pharmacovigilance suggests musculoskeletal and possible cardiac reporting signals, requiring confirmation in disease-specific cohorts [125]	No clear CRSwNP-specific ISR pattern has yet been established

## Data Availability

No new data were created or analyzed in this study.

## Abbreviations

The following abbreviations are used in this manuscript:

CRSwNP	Chronic rhinosinusitis with nasal polyps
CRS	Chronic rhinosinusitis
CRSsNP	Chronic rhinosinusitis without nasal polyps
ILC2	Type 2 innate lymphoid cells
AERD/NSAID-ERD	Aspirin-exacerbated respiratory disease/NSAID-Exacerbated Respiratory Disease
CT	Computed tomography
NPS	Nasal Polyp Score
SNOT-22	Sino-Nasal Outcome Test
MeSH	Medical Subject Headings
EPOS	European Position Paper on Rhinosinusitis and Nasal Polyps
EAACI	European Academy of Allergy and Clinical Immunology
EUFOREA	European Forum for Research and Education in Allergy and Airway Diseases
INCS	Intranasal corticosteroids
SCS	Systemic corticosteroids
ECP	Eosinophil cationic protein
MPO	Myeloperoxidase
FESS	Functional endoscopic sinus surgery
OMC	Osteomeatal complex
ARS	Acute rhinosinusitis
NOSE	Nasal Obstruction Symptom Evaluation
L-FESS	Limited-functional endoscopic sinus surgery
E-FESS	Expanded–functional endoscopic sinus surgery
IL-4Rα	Interleukin-4 receptor alpha
AIFA	Italian Medicines Agency
FcεRI	High-affinity IgE receptor
FcεRII	Low-affinity IgE receptor
IL-5Rα	Interleukin-5 receptor alpha
NK	Natural killer cells
ADCC	Antibody-dependent cell-mediated cytotoxicity
VAS	Visual Analog Scale
UPSIT	University of Pennsylvania Smell Identification Test
AE	Adverse event
SAE	Serious adverse event
N-ERD	NSAID-exacerbated respiratory disease
